# A mouse model of pulmonary *Mycobacteroides abscessus* infection

**DOI:** 10.1038/s41598-020-60452-1

**Published:** 2020-02-28

**Authors:** Emily C. Maggioncalda, Elizabeth Story-Roller, Julian Mylius, Peter Illei, Randall J. Basaraba, Gyanu Lamichhane

**Affiliations:** 1 Division of Infectious Diseases, School of Medicine, Johns Hopkins University, Baltimore, MD 21287 USA; 2 Department of Microbiology, Immunology, and Pathology, College of Veterinary Medicine and Biomedical Sciences, Colorado State University, Fort Collins, CO 80523 USA; 3 The Sidney Kimmel Comprehensive Cancer Center, Johns Hopkins University School of Medicine, Baltimore, MD 21205 USA; 4 Department of Pathology, Johns Hopkins University School of Medicine, Baltimore, MD 21205 USA

**Keywords:** Animal disease models, Pathogens

## Abstract

There is no preclinical mouse model to investigate pulmonary *Mycobacteroides abscessus* (formerly *Mycobacterium abscessus*) infection in an immunocompetent mouse strain, especially in the context of antibiotic testing and regimen development. We developed a mouse model of pulmonary *M. abscessus* infection using the aerosolized route of infection that leads to an increase in bacterial burden post- implantation and develops pathology as a result. In this mouse model, treatment with corticosteroid allows for initial proliferation and sustained *M. abscessus* pulmonary infection and permits evaluation of efficacies of antibiotics. Administration of corticosteroids that permitted higher levels of bacterial burden in the lungs were more likely to have pathology. Treatment of mice with antibiotics administered intranasally or subcutaneously significantly reduced lung *M. abscessus* burden. In addition to the reference strain, independent clinical isolates of *M. abscessus* also readily establish infection and proliferate in the lungs of mice in this model.

## Introduction

In the setting of structural lung conditions such as cystic fibrosis, bronchiectasis, and COPD, *Mycobacteroides abscessus* (formerly *Mycobacterium abscessus*) can cause chronic pulmonary infection^1,2^ that is often incurable and associated with rapid lung function decline^3–5^. There is a current interest in developing new drugs and regimens to treat *M. abscessus* infection, as the cure rate with existing antibiotics is only 30–50%^6^. While there have been many exciting demonstrations of *in vitro* potencies of antibiotics against *M. abscessus*^7–13^, it has also been established that potency observed *in vitro* for *M. abscessus* often does not translate to an equivalent clinical efficacy in the case of pulmonary infections^3^. This highlights the need for a preclinical animal model to evaluate experimental antibiotics. A mammalian model that recapitulates aerosol infection leading to progressive *M. abscessus* burden and development of lung pathology as observed in human disease would facilitate preclinical studies of antibiotic efficacy and *M. abscessus* genetic determinants for virulence.

In investigations of *Mycobacterium tuberculosis* (*M. tuberculosis*), several studies have demonstrated that aerosol infected C3HeB/FeJ mice closely mimic human pulmonary pathology^14–17^. However, when infected with *M. abscessus*, these mice are able to clear the infection^18^. Other immunocompetent mouse strains also clear the infection over time, although certain genetically manipulated immunocompromised strains were able to maintain bacterial burden^19–22^. This is a compromise in favor of perpetuating the infection, but at a loss of immune cells and inflammatory markers normally observed in response to infection. Therefore, as mice are unable to mount an immune response, this precludes evaluation of critical aspects of host-pathogen interactions and pathology development that may impact efficacy of antibiotic treatments.

We hypothesized that transient pharmacologically-induced immune suppression of immunocompetent mice such as the C3HeB/FeJ strain may allow for proliferation of *M. abscessus* during the acute stage of infection, followed by a sustained infection that leads to development of pathologic lesions. There are precedents to support this hypothesis. In the Cornell model of latent tuberculosis^23^, corticosteroid use permits proliferation of *M. tuberculosis*^24^. Corticosteroid use is also a risk factor for development of invasive infections with rapidly-growing mycobacteria^25–27^. We applied these concepts by administering corticosteroids to mice to increase their susceptibility to an initial infection with *M. abscessus*.

Recently, we demonstrated proof-of-concept of the utility of pharmacological immunosuppression of C3HeB/FeJ mice to evaluate efficacies of subcutaneously delivered dual β-lactam regimens to treat pulmonary *M. abscessus* infection^28^. However, in this previous study we did not investigate how pathology develops during steroid treatment and upon steroid treatment release. Additionally, with the recent success of liposomal amikacin for inhalation in treating refractory *Mycobacterium avium* complex (MAC) infection^29^, we also tested the utility of our model for evaluation of alternative routes of antibiotic delivery, such as intranasal administration. Finally, we assessed infection and growth profiles of six additional *M. abscessus* strains isolated from cystic fibrosis patients.

Here, we describe a mouse model of aerosolized pulmonary *M. abscessus* infection with associated lung pathology. We also demonstrate its utility in evaluating efficacy of antibiotic therapies administered via the intranasal route as well as infection with clinical strains of *M. abscessus*, both of which have not been previously described with this model.

## Methods

### Ethics

Animal procedures were performed in adherence to national and Johns Hopkins University Animal Care and Use Committee guidelines, and were approved by the Johns Hopkins University Animal Care and Use Committee (animal protocol number MO17M279).

### Bacterial strains and growth conditions

*Mycobacteroides abscessus* reference strain ATCC 19977 was used and cultured as published previously^28^. We also evaluated six clinical de-identified *M. abscessus* strains archived in the Clinical Microbiology Laboratory of the Johns Hopkins University Hospital. These strains are 5 N, 202, 214, 215, JHH4, and JHHKB and their susceptibilities to antibiotics commonly used to treat *M. abscessus* disease have been characterized^30^. Additionally, these strains were authenticated by sequencing their whole genome using Illumina 2 × 300 platform. Lungs, liver and spleen were collected aseptically and CFU enumerated; these procedures are described in more detail in the supplemental information.

### Mouse infections and treatment

Female C3HeB/FeJ mice, 4–5 weeks old, were procured from Jackson Laboratories. To achieve an implantation of ~3.0–3.5 log_10_ CFU in the lungs of a mouse, a primary *M. abscessus* culture at exponential phase, A_600nm_ of 1.00–1.20, was used to prepare a suspension by diluting to a calculated A_600nm_ of 0.1 in Middlebrook 7H9 broth prewarmed to 37 °C. Infections were performed by aerosolizing 10 mL of this suspension using a Glas-Col Inhalation Exposure System (Glas-Col, Terre Haute, Indiana), as per the manufacturer’s instructions. The infection cycle comprised of 15 minutes of pre-heat, 30 minutes of nebulization, 30 minutes of cloud decay and 15 minutes of surface decontamination.

**Figure 1:**
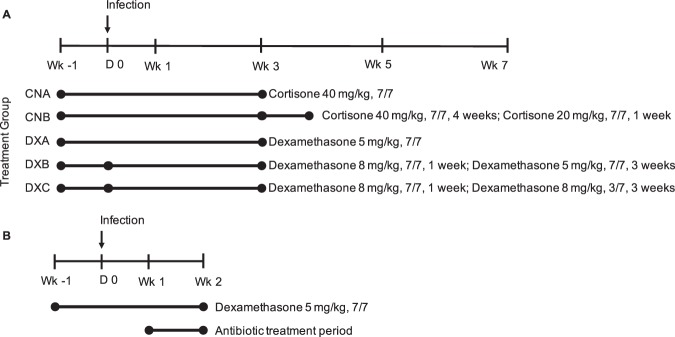
(**A**) Outline of corticosteroid dosages, dosing frequency, and duration of administration. X in X/7 indicates days of treatment per week. Treatments began one week prior to infection. The treatment group naming convention is as follows: first two letters indicate corticosteroid administered (CN = cortisone, DX = dexamethasone), third letter indicates a different treatment regimen using that corticosteroid (A or B for cortisone or A, B, or C for dexamethasone). Duration of treatment is indicated by length of the lines, with changes in regimen indicated by line segmentation at the relevant time points. As controls, mice that did not receive corticosteroids were also included. Sacrifices were performed 24 hours post infection (D0) and then at weeks 1, 3, 5 and 7 post infection. (**B**) Study and treatment outline for testing the efficacy of intranasal administration of biapenem in comparison to subcutaneous administration. Mice were sacrificed 24 hours post infection (D0) to enumerate implantation, at 1 week post infection to enumerate bacterial burden prior to antibiotic treatment period, and then at 2 weeks post infection/1 week post antibiotic treatment to evaluate treatment efficacies.

Suspensions of cortisone (Sigma-Aldrich C2755) and dexamethasone (Sigma-Aldrich D1756) were prepared immediately prior to administration in sterile 1x phosphate buffered saline, pH 7.4 (Quality Biological, 114-058-101). A 200 μL bolus of either corticosteroid was administered by subcutaneous injection in the dorsal abdominal flank using a 26 gauge syringe. Calculations for dosage were made assuming a 25 g mouse. No treatments were performed on the day of infection with *M. abscessus*. Figure 1A provides an outline of the study. More details of the study can be found in Table S1. Corticosteroid dosing strategies for this experiment were based on prior experiments with aerosolized *M. abscessus* mouse infection (Figs. S1–S3) as well as information from the literature^24,31–33^. The DXA dosing scheme was used for the clinical strain infection experiment, with sacrifices only at day 0, week 1 and week 3.

### Antibiotic delivery study treatments

All mice received 5 mg/kg dexamethasone, 7 days a week, for one week prior to and two weeks post infection with *M. abscessus*. Three groups of mice were included: a control group that did not receive antibiotic, one group that received biapenem intranasally, and the other subcutaneously. Treatment with biapenem (intranasal and subcutaneous dosing 300 mg/kg, q12) was initiated one week following infection and administered for one week. For subcutaneous injection, a 200 uL bolus of biapenem in 1x PBS was delivered twice daily via 26 G syringe. For intranasal dosing, a total of 40 uL of biapenem in 1x PBS was administered twice daily, 20 uL per nostril using 20 uL pipette, separated by 10 minutes to allow for absorption/clearance of drug. Mice were sacrificed 24 hours post infection (D0) to enumerate implantation, at 1 week post infection to enumerate bacterial burden prior to antibiotic treatment period, and then at 2 weeks post infection/1 week post antibiotic treatment to evaluate treatment efficacies. The treatment outline for this study is shown in Fig. 1B.

Details on the methods of histology preparation, pathology analysis, and statistical methods can be found in the supplemental information.

## Results

### Bacterial burden

**Figure 2:**
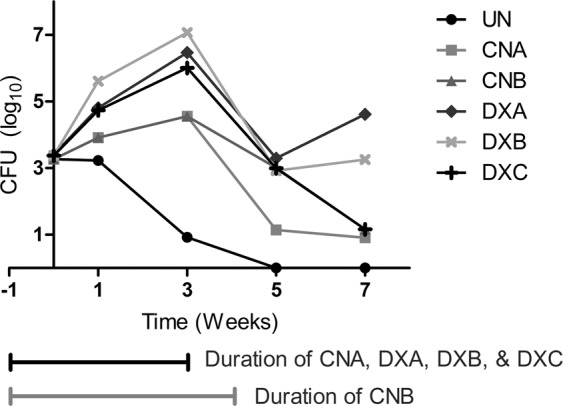
*M. abscessus* burden in the lungs of mice receiving different corticosteroid treatments. Horizontal bars designate the duration of the treatment conditions relative to sacrifice time points. Plot of mean lung log_10_ CFU.

After infection mice in all groups had an equivalent starting pulmonary implantation of ~3 log_10_ CFU of *M. abscessus*. In mice untreated with corticosteroid, *M. abscessus* lung burden remained steady at one week following infection but was gradually cleared thereafter, reaching an undetectable level at the five week time-point (Fig. 2, Table 1). In mice that received corticosteroid, *M. abscessus* lung burden increased throughout the treatment period. In the cortisone treatment groups this increase occurred at a slower rate than the dexamethasone treatment groups.

**Table 1:** *M. abscessus* burden in the lungs of mice receiving different corticosteroid treatments.

Treatment Group	Mean log_10_ CFU/Lung ± SD				
	Treatment Period			Post Treatment Period	
	D0	Wk1	Wk3	Wk5	Wk7
Untreated (UN)	3.3 ± 0.1 (n = 5)	3.2 ± 0.8 (n = 5)	0.9 ± 0.9 (n = 5)	0.0 ± 0.0 (n = 5)	0.0 ± 0.0 (n = 5)
CNA	3.3 ± 0.2 (n = 5)	3.9 ± 1.0 (n = 5)	4.6 ± 0.9 (n = 5)	1.1 ± 1.1 (n = 5)	0.9 ± 1.3 (n = 5)
CNB				3.0 ± 0.7 (n = 5)	1.2 ± 1.1 (n = 5)
DXA	3.4 ± 0.1 (n = 5)	4.8 ± 0.7 (n = 5)	6.5 ± 0.5 (n = 5)	3.3 ± 0.5 (n = 5)	4.6 ± 0.2 (n = 4)
DXB	3.4 ± 0.2 (n = 4)	5.6 ± 0.3 (n = 5)	7.1 ± 1.4 (n = 5)	2.9 ± 1.0 (n = 5)	3.3 ± 2.1 (n = 2)
DXC		4.7 ± 1.0 (n = 5)	6.0 ± 0.9 (n = 5)	3.0 ± 0.4 (n = 5)	1.2 ± 1.1 (n = 5)

All groups began with equivalent lung implantation burdens. D0 refers to day mice were infected with *M. abscessus*, and WkX refers to X weeks following infection.

Following cessation of corticosteroid administration, *M. abscessus* lung burden declined in all groups with the exception of the DXA group where we observed a slight increase in CFU between 5 and 7 week time-points. In the cortisone treated mice, group CNB, which received an additional week of half-dose cortisone taper, *M. abscessus* lung burden was ~2 log_10_ higher at the week 5 time-point compared to CNA which did not have a steroid taper. However, by the 7 week time-point, *M. abscessus* was almost completely cleared by both groups of mice.

To assess the effect of dexamethasone on *M. abscessus* lung burden, we included three different groups with distinct dosing regimens (Fig. 1A). Mice in group DXA, which received a daily dose of 5 mg/kg dexamethasone, exhibited a robust growth of *M. abscessus* in the lungs during the treatment phase and lowest rate of clearance during treatment discontinuation phase.

We observed the most rapid *M. abscessus* proliferation, highest *M. abscessus* burden (Fig. 2, Table 1) and higher levels of morbidity in DXB group mice necessitating sacrifice of two mice at 3.5 week time-point and one mortality at the week 4 time-point. This group received the highest overall dexamethasone dose. We had one mortality at the 3.5 week time-point in the DXA group as well. Mice in the DXC group, which received intermittent dexamethasone following *M. abscessus* infection, demonstrated a steep decline in CFU once treatment was discontinued.

To explore the extra-pulmonary bacterial burden that may occur as part of these corticosteroid regimens, we enumerated the *M. abscessus* burden in the liver and spleen as well. *M. abscessus* burden at these sites also increased over time (Tables S2 and S3), although was variable across and within groups. The peak disseminated *M. abscessus* burden occurred at week 5 post-infection, after the period of peak bacterial burden in the lungs and once all corticosteroid treatments had ceased (Fig. S4).

### Pathology

**Figure 3:**
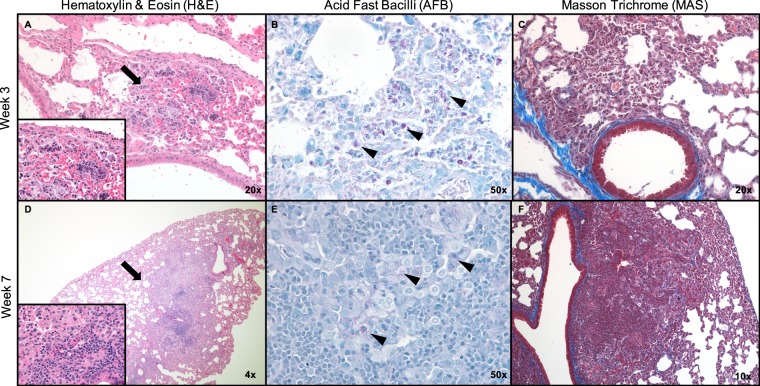
Lung histology images from the DXA treatment group examined with Hematoxylin & Eosin (H&E), Acid fast bacilli (AFB) and Masson Trichrome (MAS) stains. Lesions observed in H&E are designated with arrows. AFB stains *M. abscessus* purple/red and some of the stained rods are indicated using arrowheads. MAS stains collagen blue to investigate any irregular collagen deposition and fibrosis. At week 3 there was one small lesion on H&E (**A**) with high bacterial burden observable (**B**), low levels of immune infiltrate and no fibrosis (**C**). At week 7 we observed an organized histiocytic granuloma, with lymphocytic infiltrate observable at higher magnification (inset, **D**). There were low numbers of bacilli present which all appeared to be intracellular (**E**) and no fibrosis (**F**). (H&E insets 50x magnification).

The number and proportion of lesions in the lungs of mice in cortisone, dexamethasone or untreated mice are summarized in Table 2. Prior to the week 3 time-point, we were unable to observe any evidence of lung pathology in all treatment groups. At the week 3 time-point, only mice in the DXA group developed lesions in their lungs. The lesion observed at this time-point was small but harbored high numbers of *M. abscessus* bacilli (Fig. 3A–C) consistent with CFU for this time point (Fig. 2). It is notable that compared to all other treatment groups, DXA had the most consistent presence of pathology in the standard section at the week 5 and week 7 time-points. One mouse at week 5 developed a large necrotic lesion with abscesses, which included large numbers of bacilli and early organizing fibrosis observable on Masson’s trichrome sections (Fig. S5). Large numbers of neutrophils were also identified in this lesion as well as macrophages and lymphocytes. Other standard sections from this time point had more mild pathology, more closely resembling observations at week 3. At week 7, one individual had a granuloma containing macrophages and lymphocytes, but less necrosis was observed and neutrophils were less common. Bacilli were also present in the lesion, but were sparse compared to the necrotic lesions, and there was no organizing fibrosis (Fig. 3D–F).

**Table 2:** Quantification of lesions from a standardized section of lung tissue.

Time point/ Treatment	Wk3	Avg % Lesion	Wk5	Avg % Lesion	Wk7	Avg % Lesion
UN	0/2	—	0/5	—	0/5	—
CNA	0/2	—	1/5	2.54	1/5	0.93
CNB			0/5	—	1/5	4.16
DXA	1/2	1.43	3/5	23.90	2/4	4.37
DXB	0/2	—	3/5	23.70	1/2	69.84
DXC	0/2	—	3/5	3.90	1/5	5.58

X/Y indicates the number of individuals sampled at the time point that had a lesion in the standardized section. “Avg % Lesion” is the average percentage of the section that was lesion, across the sampled mice that had lesions. No pathology was observed prior to the week 3 timepoint.

The lungs of mice in the DXC group had pathology similar in appearance to the non-necrotic lesions in the DXA mice but were infrequent (Fig. S6). In contrast to the controlled histiocytic granulomas observed in DXA and DXC, DXB trended towards necrosis with large numbers of neutrophils and abundant intracellular (in macrophages) and extracellular bacilli (Fig. S7). Neutrophilia was especially apparent in one mouse in which vasculature was occluded with neutrophils and fewer lymphocytes. Mice in both cortisone administered groups had a low lesion burden compared to dexamethasone treatment groups. Few gross pathological lesions could be observed on the lungs with the exception of moribund mice.

### Application in antibiotic evaluation

**Figure 4:**
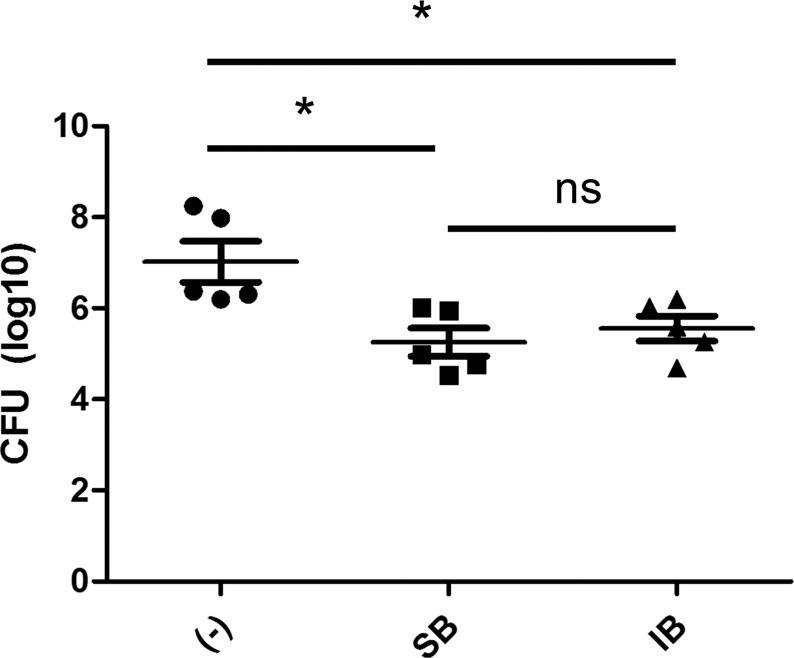
Investigation into intranasal administration of biapenem in the DXA model of *M. abscessus* infection. Mean ± SD lung log_10_ CFU in mice that did not receive biapenem (−), subcutaneously delivered biapenem (SB), and intranasally delivered biapenem groups (IB), after one week of treatment. (*p < 0.05).

Biapenem is a broad-spectrum β-lactam of the carbapenem subclass^34^ with demonstrated *in vitro* activity against *M. abscessus*, with an MIC of 16 μg/mL^35^. It is not currently FDA approved, but is approved for use in Japan. To evaluate the utility of the mouse model to test antibiotics via alternate routes of administration we immunosuppressed the mice using the DXA regimen and treated mice with biapenem administered either subcutaneously or intranasally. Compared to the control group that did not receive antibiotic, the lung *M. abscessus* burden was reduced by ~2 log_10_ at the completion of one week of twice daily treatment with biapenem. The efficacy of biapenem in reducing *M. abscessus* lung burden was indistinguishable between the two routes of administrations (Fig. 4). This and our previous study demonstrate proof-of-concept of utility of this mouse model for various routes of delivering antibiotics against *M. abscessus*.

### Infection with clinical *M. abscessus* isolates

**Figure 5:**
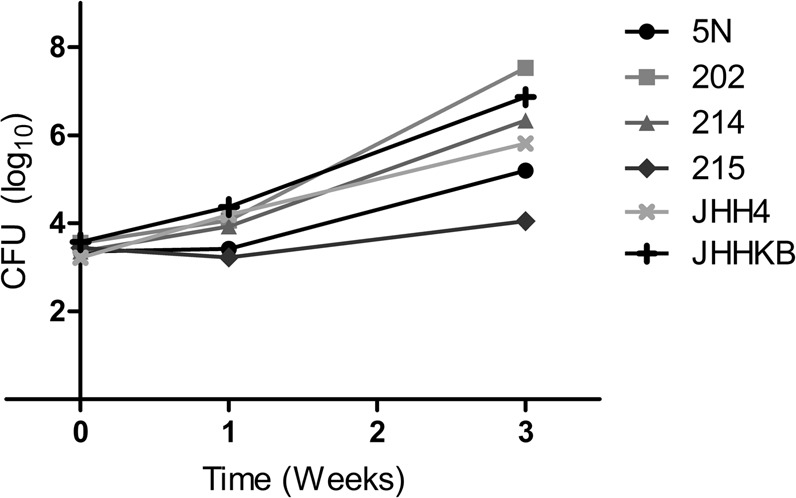
Burden of six clinical *M. abscessus* isolates in the lungs of mice. Plot of mean lung log_10_ CFU.

Lung burdens of all six isolates one day following aerosolization, a measure of their ability to implant and establish infection, were similar to that of the reference strain ATCC 19977 (Fig. 5, Tables S4 and 1 for ATCC 19977 data). At one week following infection strains 202, 214, JHH4, and JHHKB had increases in average log_10_ CFU, while strains 5 N and 215 appeared to be stable over this time period. At three weeks following infection the bacterial burden with strains 202, 214, JHH4, and JHHKB continued to increase. Strains 5 N and 215 had a modest increase in average log_10_ CFU at this time point, but with a large variability in the strain 215 group due to one mouse having no CFU isolated from the lung homogenate at this time point.

## Discussion

There are currently no FDA approved antibiotics to treat *M. abscessus* pulmonary disease and recommended treatment regimens are largely ineffective, necessitating a minimum treatment duration of at least 12 months following sputum culture conversion^3,36^, often with cytotoxic antibiotics that are poorly-tolerated by patients. The need to accelerate and improve preclinical testing of novel antibiotics and regimens for *M. abscessus* pulmonary infections is urgent. Our aim was to meet the need for an animal model that more closely recapitulated pulmonary *M. abscessus* disease in humans so that it could be used as a tool to study drug efficacy against *M. abscessus*. As mouse models have contributed extensively to the study of pulmonary disease by another mycobacterium, *M. tuberculosis*^15–17,37^, we again leveraged the mouse to develop a model of pulmonary *M. abscessus* infection that utilized the aerosol route for infection, led to an increase in lung bacterial burden after implantation, and resulted in the development of relevant pulmonary pathology.

Administration of the corticosteroids cortisone or dexamethasone led to an increase in lung *M. abscessus* burden post-implantation across all dosing strategies. However, the rate of increase in *M. abscessus* burden was slower in mice that received cortisone, resulting in lower peak burden and corresponding lack of lung pathology. In prior experiments with cortisone treatment that reached a higher bacterial burden, pathology was observed (Fig. S2E). Further investigation of pathology in mice treated with cortisone compared to dexamethasone is warranted to determine whether one corticosteroid is preferable for relevant pathology development, and highlights the need for investigations into other immunomodulatory agents as well.

Following cessation of corticosteroid administration, we observed a decrease in *M. abscessus* burden in the lungs across all groups at the week 5 time-point. An enhanced immune response post-steroid removal in the form of increased infiltration of neutrophils and lymphocytes was consistently observed. Corticosteroids like dexamethasone are known to have many immune-modulatory effects that vary by cell type. The relationship between glucocorticoids and T-lymphocytes is complex, but there appears to be a consensus in the literature that glucocorticoids lead to thymocyte apoptosis, with subsequent decrease in immature T-cell counts^38–41^ and skewing of the T-cell response toward Th2^41–43^. In addition, we hypothesize that corticosteroid treatment in mice reduced migration of neutrophils from the blood into the lungs, coupled with reductions in superoxide and reactive oxygen species, which may have allowed *M. abscessus* to proliferate unimpaired^44–47^. No animal model is able to comprehensively simulate all aspects of human infection and pathology, nor does any one model meet all investigative goals within. As investigations into host-directed therapies and immunomodulatory therapies move forward against *M. abscessus* infections^48^, other models that do not have immune system manipulation as a limitation are necessary.

We found administration of 5 mg/kg dexamethasone once daily (DXA) to be the most promising dosing strategy, as this dosing regimen was well-tolerated in the mice and achieved the desired high level of lung *M. abscessus* burden with extended duration of infection. The duration of persistence of pulmonary *M. abscessus* infection using the DXA regimen following discontinuation of dexamethasone, as well as the mechanistic basis for the observed rebound in infection were beyond the scope of the current proof-of-concept study. Regardless, the extended period of lung *M. abscessus* burden provides valuable extra time for the evaluation of efficacy of antibiotics. We undertook additional proof-of-concept studies of the DXA dosing strategy with *M. abscessus* clinical isolates and in Balb/c mice. Clinical isolates demonstrated variable ability to replicate *in vivo* compared to the reference strain, while Balb/c mice developed a similar increase in lung *M. abscessus* burden post-implantation (Fig. S8). Pathology development and CFU trends following steroid cessation were not evaluated in these short studies, but these data suggest that the DXA dosing strategy may be utilized in multiple murine strains and with other strains of *M. abscessus*.

In our prior studies, persistent use of steroids did not produce defined granulomas characterized by organized migration and localization of immune cells and instead resulted in a necrotizing pneumonia (Fig. S1C–H). By contrast, upon removal of corticosteroids, we observed pathology ranging from small granulomas to extensive necrosis and abscessation (Fig. S2E). In the current study, the pathology observed in dexamethasone-administered groups followed a general pattern of lesions with higher bacterial burdens being more necrotic and neutrophil-rich with associated macrophages, lymphocytes, and organizing fibrosis; whereas lesions with lower bacterial burdens had less neutrophilic infiltrate and little fibrosis, but still contained foamy macrophages and lymphocytic infiltrate. This suggests that pathologic lesions resulting from *M. abscessus* infection may exhibit similar lesion typology as has been described for *M. tuberculosis* infections in C3HeB/FeJ mice^16^. It is worthwhile to note that mice not found to have lesions in standardized lung sections does not preclude the presence of lesions in other levels of the lung; however, in order to reduce bias in selection of lesions for analysis, this method of histology preparation was performed. An extensive characterization of pathology and associated immune response and dynamics of lung *M. abscessus* burden over longer durations was beyond the scope the current study.

The model described in this study recapitulates salient hallmarks of pulmonary *M. abscessus* disease in humans such as the natural route of infection, proliferation of *M. abscessus* and persistence of infection resulting in organized lesions with involvement of macrophages, neutrophils, and lymphocytes. This model allows for evaluation of efficacies of antimicrobial chemotherapies that may be administered via various routes and therefore addresses the current need for an animal model for preclinical development of drugs and regimens to treat *M. abscessus* infection. Evidence that this model is amenable to the use of more antibiotic resistant clinical isolates also increases its functionality for preclinical drug testing. As this was a proof-of-concept study, additional studies are warranted to further characterize the immune pathology and infection dynamics over a longer duration.

## Supplementary information

Supplemental Figures and Tables.

Supplemental Methods.
