# Supplementary material for: A mouse model of pulmonary Mycobacteroides abscessus infection
Source: Sci Rep. 2020 Feb 28;10:3690. doi: 10.1038/s41598-020-60452-1 (PMC7048719; doi:10.1038/s41598-020-60452-1)
Supplement: Supplementary file 1 — Supplemental Figures and Tables. [file 41598_2020_60452_MOESM1_ESM.pdf]

TITLE: A mouse model of pulmonary *Mycobacteriodes abscessus* infection

AUTHORS: Emily C Maggioncalda[1], Elizabeth Story-Roller[1], Julian Mylius[2], Peter Illei[3,4], Randall J. Basaraba[2], Gyanu Lamichhane*[1]

AFFILIATIONS: 1) Division of Infectious Diseases, School of Medicine, Johns Hopkins University, Baltimore, MD, United States 2) Department of Microbiology, Immunology, and Pathology, College of Veterinary Medicine and Biomedical Sciences, Colorado State University, Fort Collins, Colorado, United States 3) The Sidney Kimmel Comprehensive Cancer Center, Johns Hopkins University School of Medicine, Baltimore, Maryland, United States 4) Department of Pathology, Johns Hopkins University School of Medicine, Baltimore, Maryland, United States

SUPPLEMENTAL FIGURES & TABLES

Groups/Timepoint	D0	Wk1	Wk3	Wk5	Wk7	Total
Infected						
Untreated (UN)	5	5	5	5	5	25
CNA (7/7 40 mg/kg x 4 wks)	5	5	5	5	5	25
CNB (7/7 40 mg/kg x 4 wks → 7/7 20 mg/kg x 1 wk)				5	5	10
DXA (7/7 5 mg/kg x 4 wks)	5	5	5	5	5	25
DXB (7/7 8 mg/kg x 1 wk → 7/7 5 mg/kg x 3 wks)	5	5	5	5	5	25
DXC (7/7 8 mg/kg x 1 wk → 3/7 8 mg/kg x 3 wks)		5	5	5	5	20
Uninfected Pathology Controls						
Untreated (UN)	1	1	1	1	1	5
CNA (7/7 40 mg/kg x 4 wks)	1	1	1	1	1	5
CNB (7/7 40 mg/kg x 4 wks → 7/7 20 mg/kg x 1 wk)				1	1	2
DXA (7/7 5 mg/kg x 4 wks)	1	1	1	1	1	5
DXB (7/7 8 mg/kg x 1 wk → 7/7 5 mg/kg x 3 wks)	1	1	1	1	1	5
DXC (7/7 8 mg/kg x 1 wk → 3/7 8 mg/kg x 3 wks)		1	1	1	1	4

Table S1. Scheme of corticosteroid dosing study in *Mab* pulmonary mouse infection (X/7 indicates "X" treatment days per week) with mouse numbers per group and time point. CN = Cortisone, DX = Dexamethasone. One mouse in the DXB group died of unknown causes prior to infection.

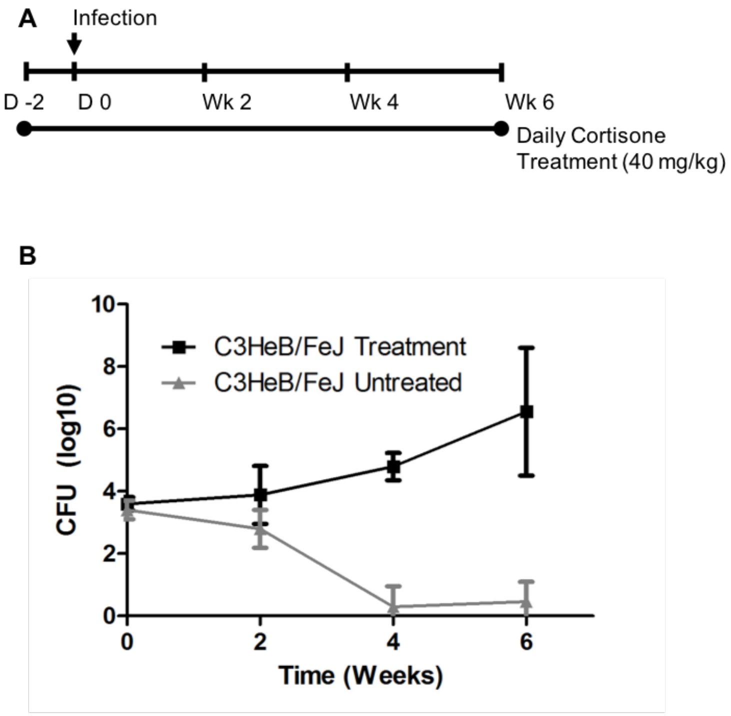

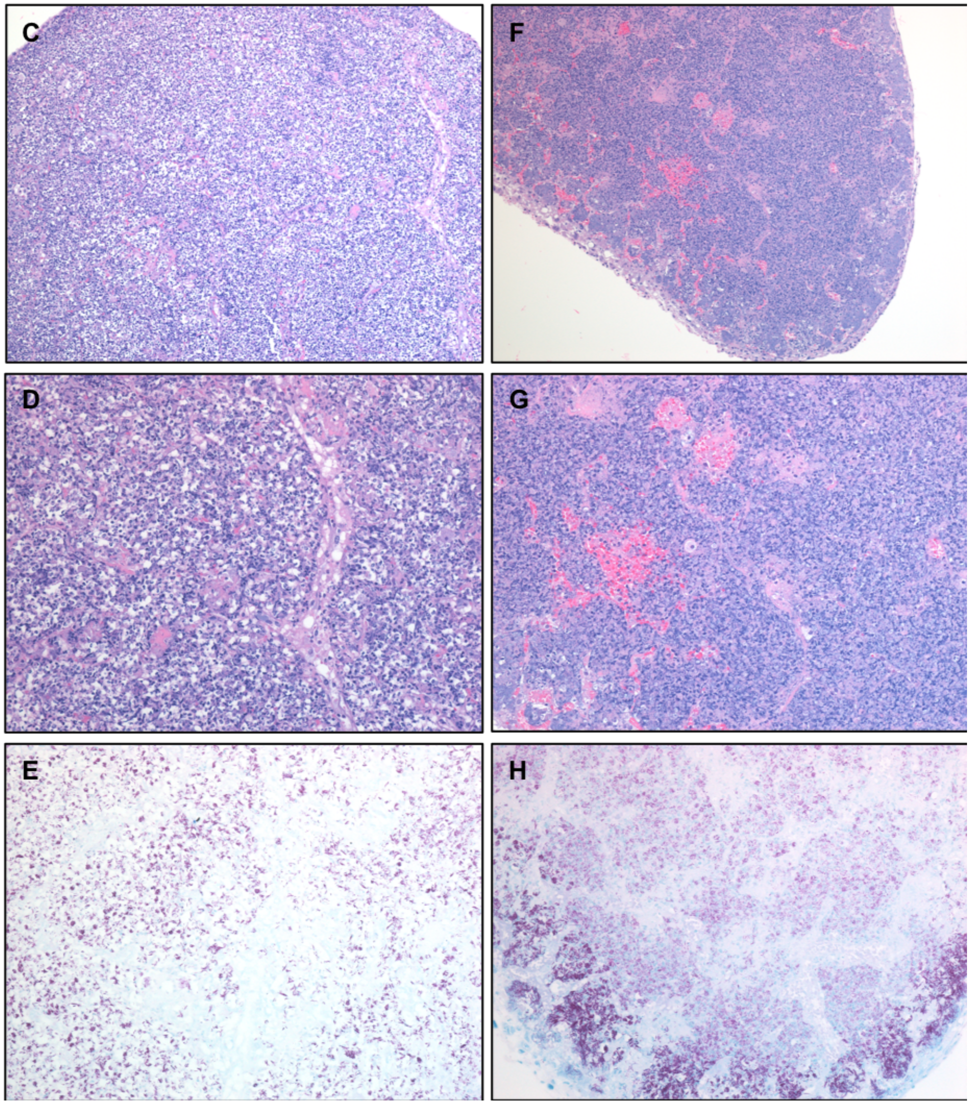

Figure S1. Initial investigation of cortisone immunosuppression in an aerosolized infection of *Mab* in C3HeB/FeJ mice (*A*) Study outline. Half of the mice received cortisone treatment, half were left untreated. (*B*) Mean ± SD $\log_{10}$ CFU of treated and untreated mice, n=5. (*C-H*) Histology from one individual that had a large lesion on gross pathology. (*C*), (*D*), (*F*) and (*G*) are H&E stained, (*E*) and (*H*) are AFB stained. Magnification of (*C*) & (*F*) is 10x. Magnification of (*D*), (*E*), (*G*) and (*H*) are 20x.

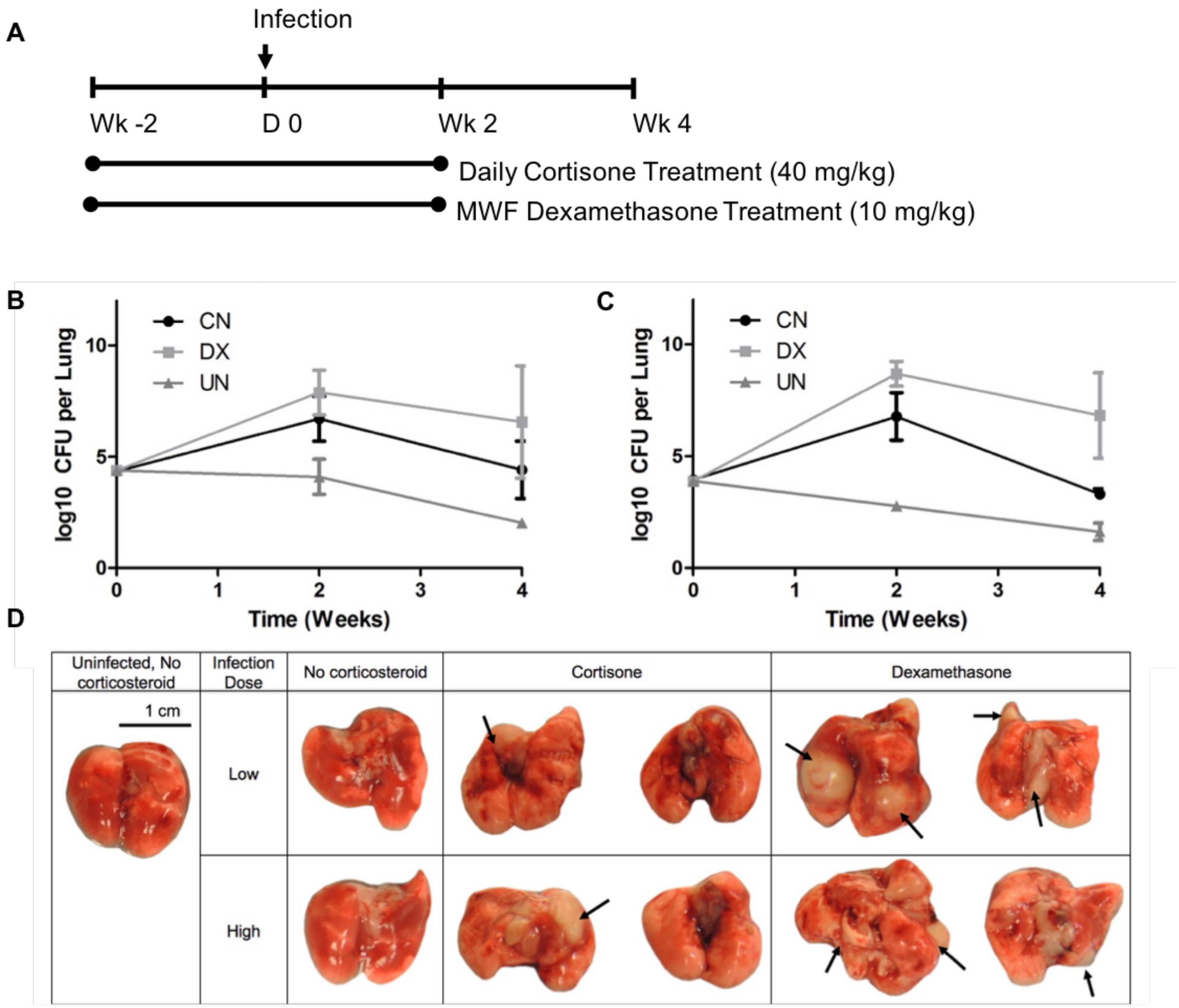

Figure S2. Comparison of use of cortisone and dexamethasone immunosuppressive regimens with removal of steroid treatment and investigation into pathology development. (*A*) Study outline. For this study two infections were performed. The first was a "low dose" infection, using a calculated OD of 0.1 for implantation. The second was a "high dose" infection, using a calculated OD of 1.0. Mean ± SD lung $\log_{10}$ CFU (n=5) for the "high" (*B*) and "low" (*C*) dose infections. Representative gross pathology at week 4, arrows denote gross lesions (*D*). Histopathology from sampled lungs at week 4 (*E*).

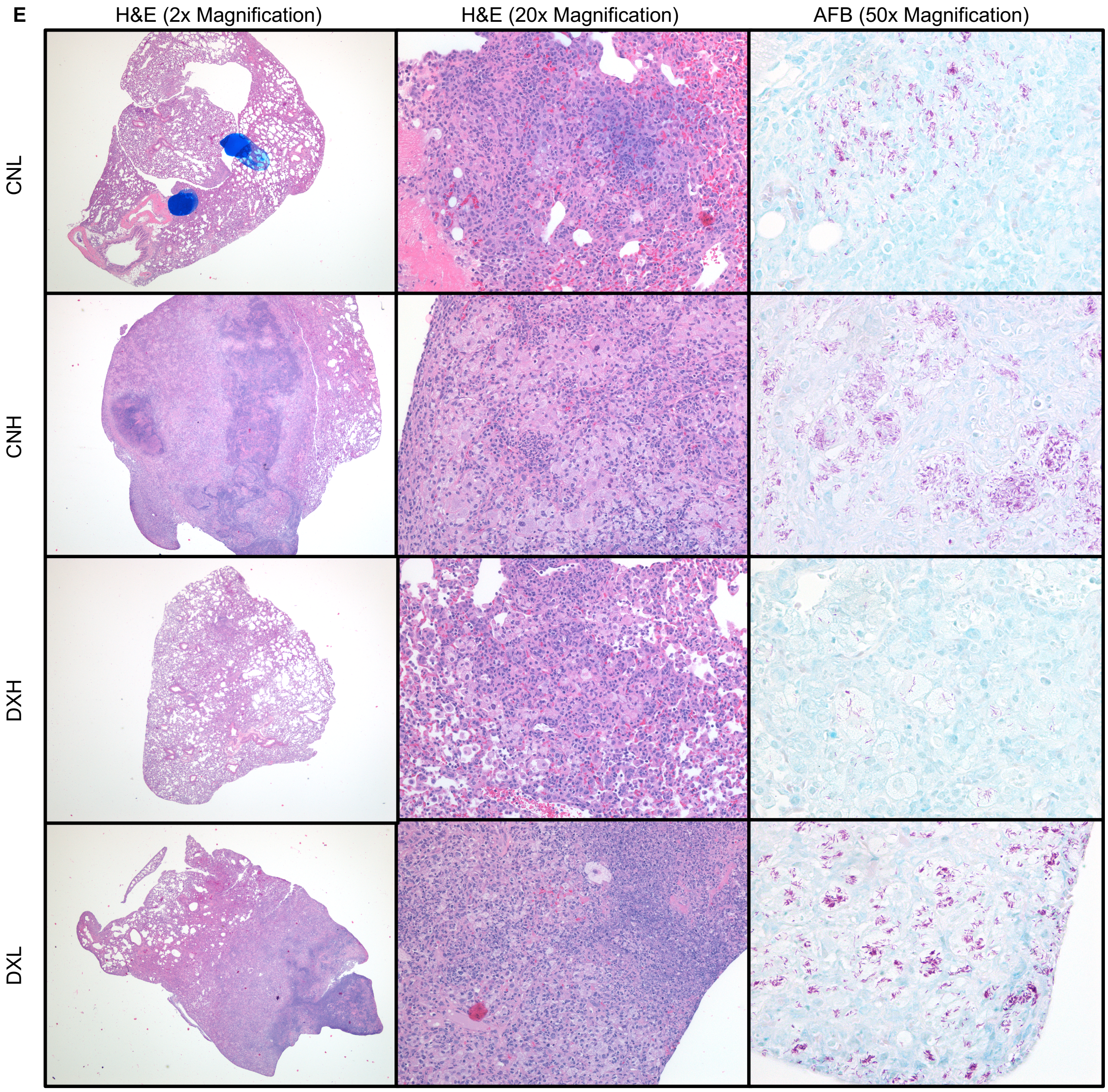
E
H&E (2x Magnification)
H&E (20x Magnification)
AFB (50x Magnification)
CNL
CNH
DXH
DXL

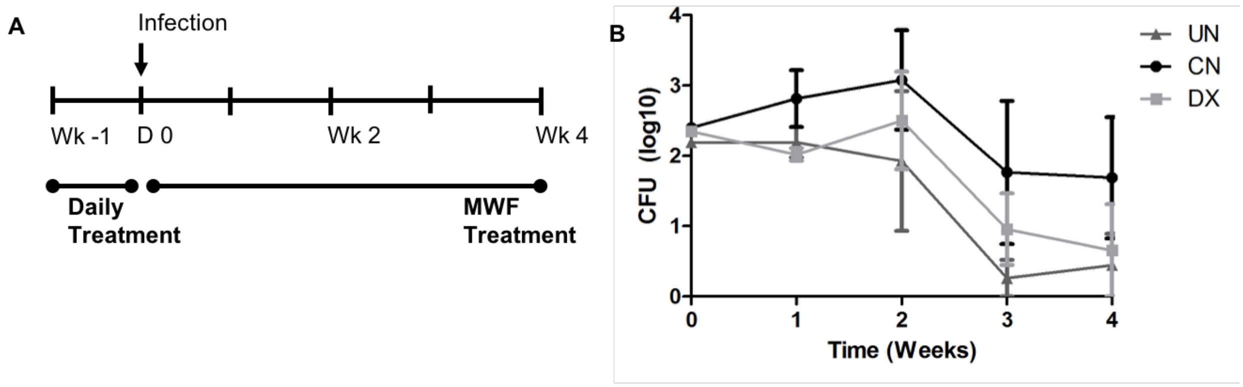

Figure S3. Investigation into low dose infection and low dose steroid treatment in aerosolized *Mab* infection. (*A*) Treatment outline. Infection used a culture to a calculated OD of 0.01. The cortisone (CN) treatment dose was 40 mg/kg. The dexamethasone (DX) treatment dose was 2 mg/kg. (*B*) Mean ± SD lung log_{10} CFU, n=5. This study undertook an effort to perform a low burden implantation with lower dose steroids and less frequent dosing. A low implantation was achieved, but the altered treatment regimens did not allow for progression of infection.

	Mean log_{10} CFU/Liver ± SD				
Treatment Group	D0	Wk1	Wk3	Wk5	Wk7
Untreated (UN)	0.0±0.0 (n=5)	0.0±0.0 (n=5)	0.0±0.0 (n=5)	0.0±0.0 (n=5)	0.0±0.0 (n=5)
CNA	0.0±0.0 (n=5)	0.0±0.0 (n=5)	0.0±0.0 (n=5)	0.0±0.0 (n=5)	1.0±1.4 (n=5)
CNB				1.0±1.4 (n=5)	0.3±0.8 (n=5)
DXA	0.0±0.0 (n=5)	0.6±1.4 (n=5)	1.5±2.1 (n=5)	2.3±1.5 (n=5)	1.9±1.5 (n=4)
DXB	0.0±0.0 (n=4)	0.0±0.0 (n=5)	2.6±2.1 (n=5)	3.3±1.5 (n=5)	1.9±2.7 (n=2)
DXC		0.0±0.0 (n=5)	2.3±2.2 (n=5)	3.0±0.5 (n=5)	1.4±1.9 (n=5)

Table S2. *M. abscessus* burden in the livers of mice receiving different corticosteroid regimens. D0 refers to day mice were infected with *Mab*, and WkX refers to X weeks following infection.

<table>
<tr><th></th><th colspan="5">Mean $\log_{10}$ CFU/Spleen ± SD</th></tr>
<tr><th>Treatment Group</th><th>D0</th><th>Wk1</th><th>Wk3</th><th>Wk5</th><th>Wk7</th></tr>
<tr><td>Untreated (UN)</td><td>0.0±0.0 (n=5)</td><td>0.0±0.0 (n=5)</td><td>0.0±0.0 (n=5)</td><td>0.0±0.0 (n=5)</td><td>0.0±0.0 (n=5)</td></tr>
<tr><td>CNA</td><td rowspan="2">0.0±0.0 (n=5)</td><td rowspan="2">0.0±0.0 (n=5)</td><td rowspan="2">0.0±0.0 (n=5)</td><td>0.3±0.7 (n=5)</td><td>0.7±1.1 (n=5)</td></tr>
<tr><td>CNB</td><td>0.9±1.2 (n=5)</td><td>0.4±0.9 (n=5)</td></tr>
<tr><td>DXA</td><td>0.0±0.0 (n=5)</td><td>0.3±0.6 (n=5)</td><td>0.8±1.1 (n=5)</td><td>1.9±1.3 (n=5)</td><td>1.8±1.3 (n=4)</td></tr>
<tr><td>DXB</td><td rowspan="2">0.0±0.0 (n=4)</td><td>0.0±0.0 (n=5)</td><td>1.3±1.8 (n=5)</td><td>2.7±1.7 (n=5)</td><td>2.1±1.6 (n=2)</td></tr>
<tr><td>DXC</td><td>0.0±0.0 (n=5)</td><td>1.5±1.5 (n=5)</td><td>2.7±0.3 (n=5)</td><td>1.4±1.4 (n=5)</td></tr>
</table>

Table S3. *M. abscessus* burden in the spleen of mice receiving different corticosteroid regimens. D0 refers to day mice were infected with *Mab*, and WkX refers to X weeks following infection.

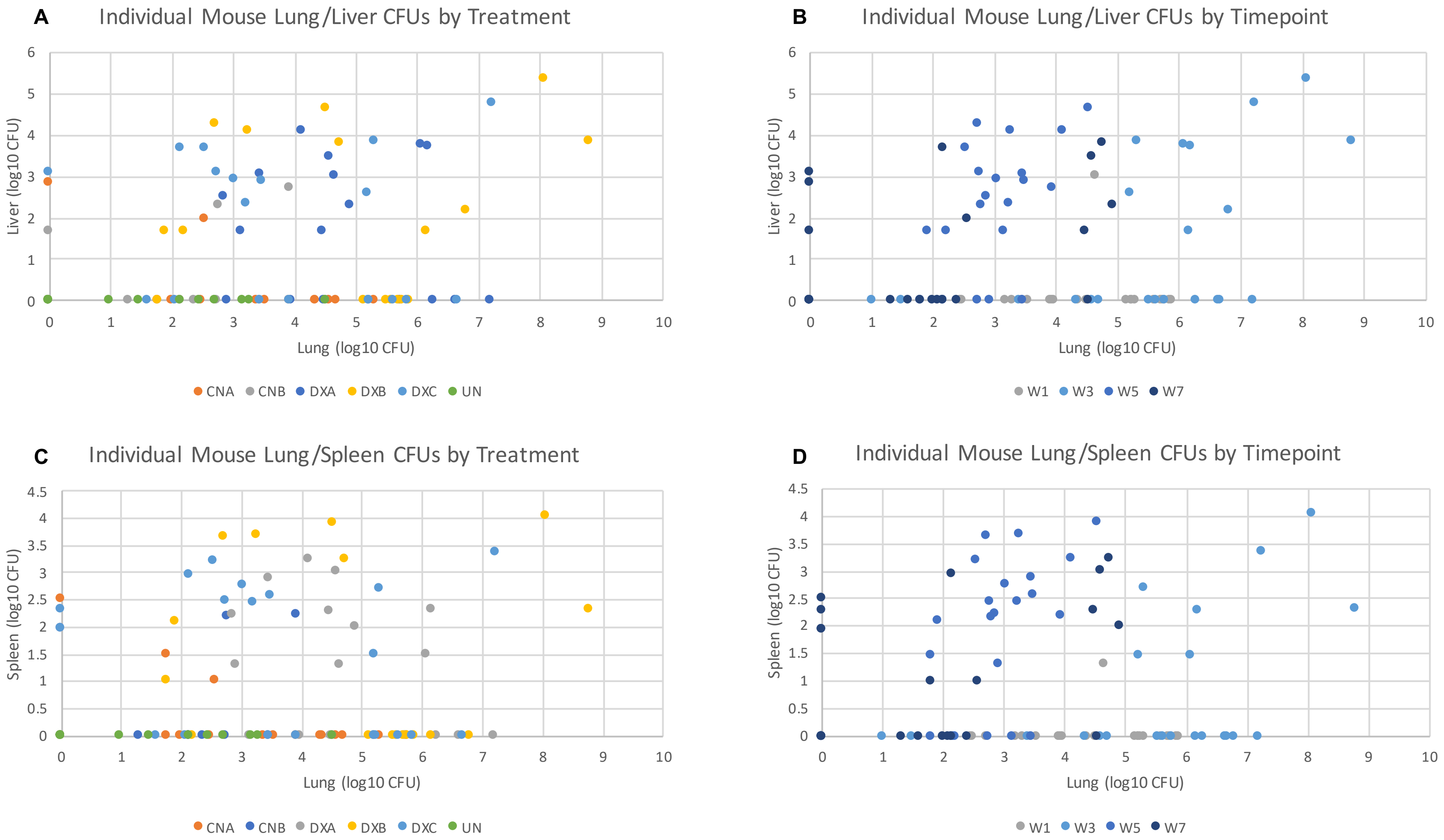

Figure S4. Analysis of associations between mouse lung *M. abscessus* CFU and either liver or spleen *M. abscessus* CFU indicated that the peak lung CFU and duration of infection appeared to be larger factors of dissemination than any treatment group. (*A*) Lung and liver log_{10} CFUs by treatment condition. (*B*) Lung and liver log_{10} CFUs by time of sacrifice. (*C*) Lung and spleen log_{10} CFUs by treatment condition. (*D*) Lung and spleen log_{10} CFUs by time of sacrifice.

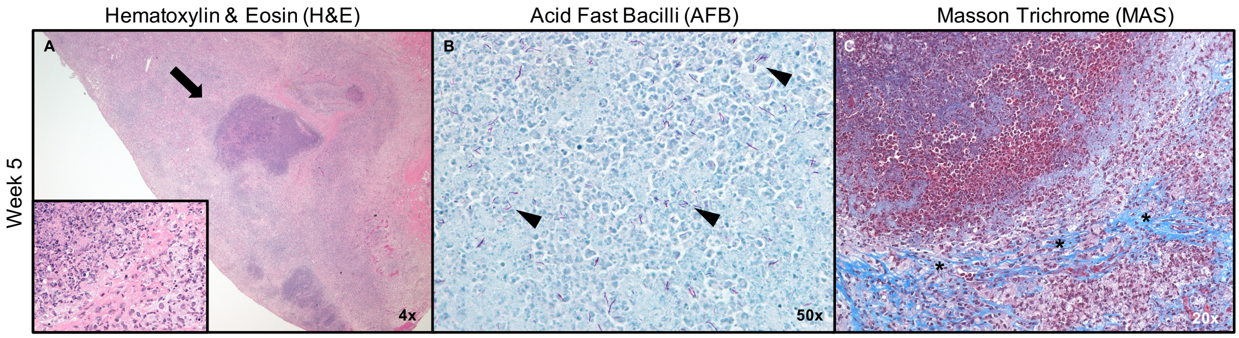

Figure S5. Lung histology images from the DXA treatment group examined with Hematoxylin & Eosin (H&E), Acid fast bacilli (AFB) and Masson Trichrome (MAS) stains. Lesions observed in H&E are designated with arrows. AFB stains *M. abscessus* purple/red and some of the stained rods are indicated using arrowheads. MAS stains collagen blue to investigate any irregular collagen deposition and fibrosis, which is indicated by an asterix. At week 5 one mouse presented with widespread necrosis with localized abscessus (*A*), high extracellular bacterial burden with neutrophilic infiltrate (*B*) and organizing fibrosis around these abscesses (*C*).

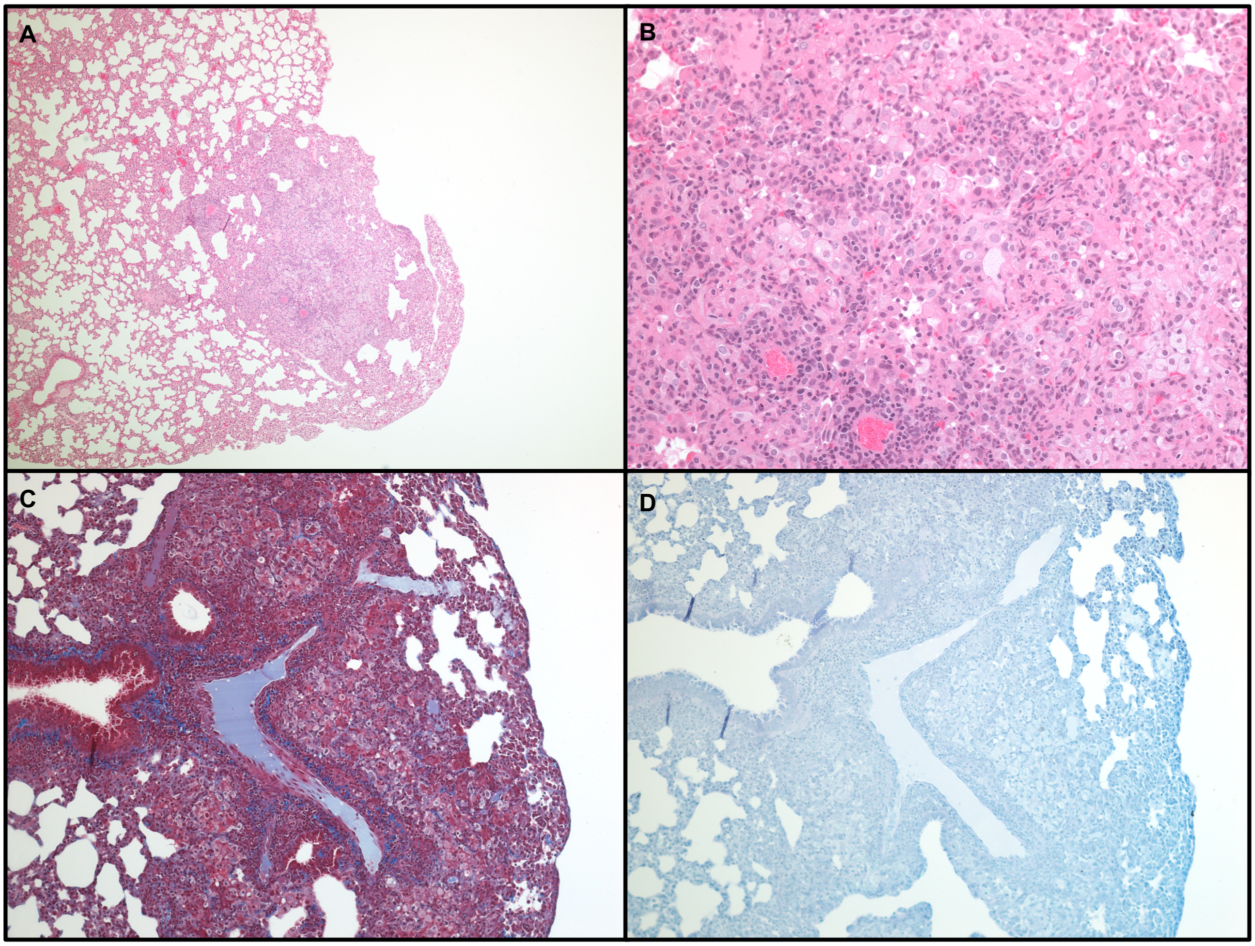

Figure S6. Representative images of the pathology observed in the DXC treatment group. Organized histiocytic granuloma with lymphocytic infiltrate is observable on H&E at low (*A*) and high power (*B*) (Magnification 4x and 20x respectively). No fibrosis observable on Masson Trichrome (*C*, magnification 10x). No bacilli observable on Acid Fast on high power magnification (*D*, low power magnification 10x).

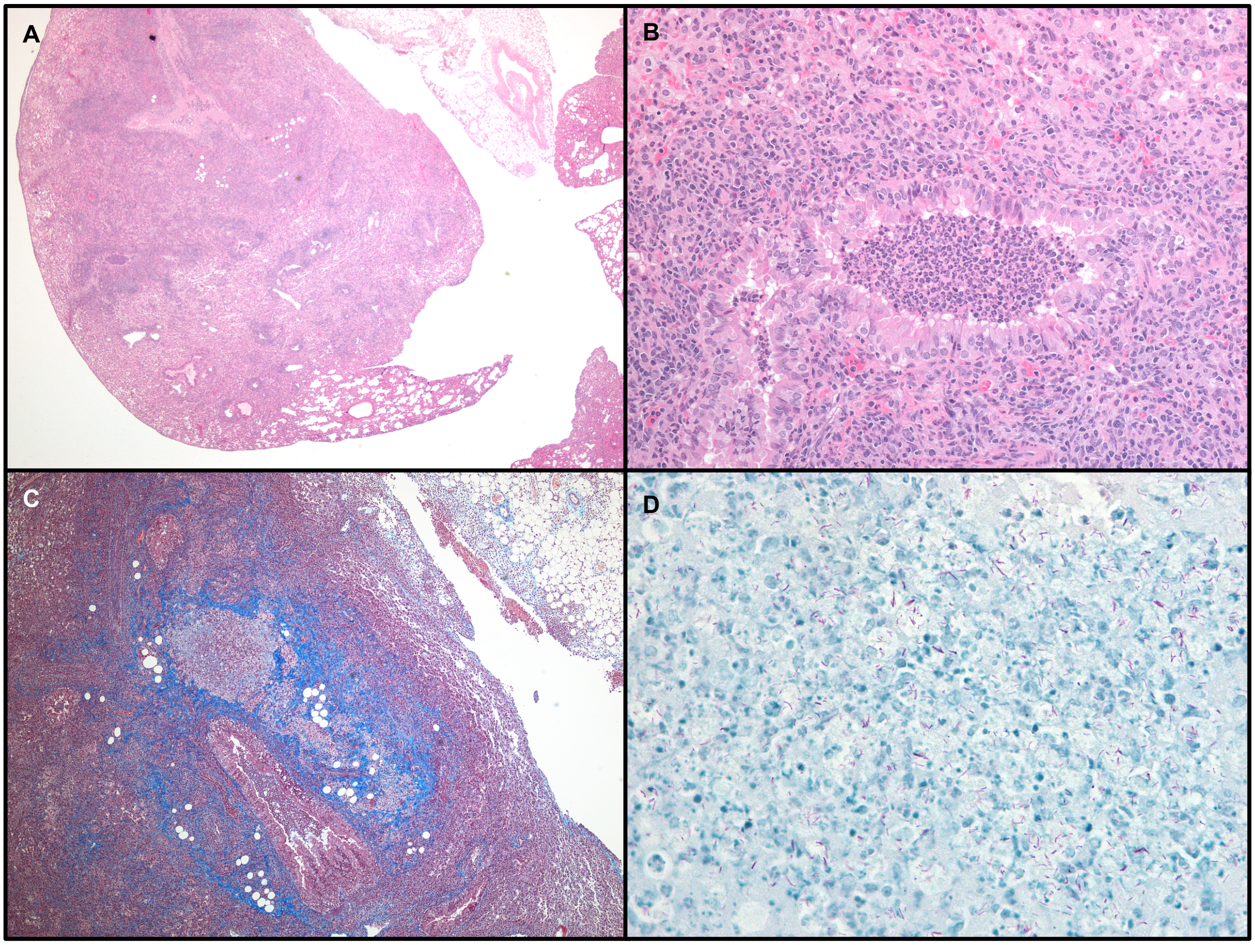

Figure S7. Representative images of the pathology observed in the DXB treatment group. Widespread necrosis with histiocytic, lymphocytic, and neutrophilic infiltrate are observable on H&E at low (*A*) and high power (*B*) (Magnification 2x and 20x respectively). Fibrosis observable around an abscess on Masson Trichrome (*C*, magnification 4x). Large numbers of extracellular bacilli observable on Acid Fast stain (*D*, magnification 50x).

	Mean $\log_{10}$ CFU/Lung ± SD		
Strain	D0	Wk1	Wk3
5N	3.4±0.1 (n=5)	3.4±0.4 (n=5)	5.2±0.8 (n=5)
202	3.5±0.1 (n=5)	4.1±0.6 (n=5)	7.5±0.6 (n=5)
214	3.4±0.0 (n=5)	3.9±0.7 (n=5)	6.3±0.8 (n=5)
215	3.4±0.1 (n=5)	3.2±0.3 (n=5)	4.0±2.3 (n=5)
JHH4	3.2±0.2 (n=5)	4.2±0.7 (n=5)	5.8±1.0 (n=5)
JHHKB	3.6±0.1 (n=5)	4.4±0.5 (n=5)	6.9±0.9 (n=5)

Table S4. Burden of six different clinical isolates of *M. abscessus* in the lungs of mice. D0 refers to day mice were infected with *Mab*, and WkX refers to X weeks following infection.

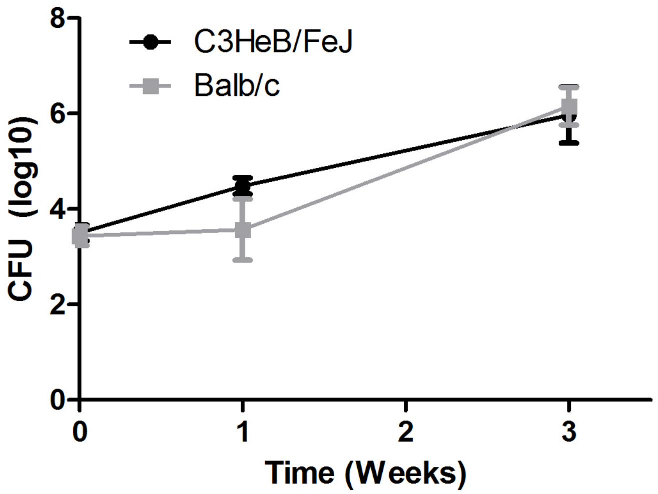

Figure S8. Investigation of DXA model of *Mab* pulmonary infection in Balb/c mice. Mean ± SD lung $\log_{10}$ CFU of DXA regimen treated C3HeB/FeJ and Balb/c mice, with the modification of 2 weeks pre-treatment instead of one. Over a period of 3 weeks the bacterial burden increased in Balb/c at the same rate as observed in C3HeB/FeJ mice. C3HeB/FeJ n=4 for all timepoints, Balb/c n=5 for all timepoints
