# Supplementary material for: A mouse model of pulmonary Mycobacteroides abscessus infection
Source: Sci Rep. 2020 Feb 28;10:3690. doi: 10.1038/s41598-020-60452-1 (PMC7048719; doi:10.1038/s41598-020-60452-1)
Supplement: Supplementary file 2 — Supplemental Methods. [file 41598_2020_60452_MOESM2_ESM.pdf]

TITLE: A mouse model of pulmonary *Mycobacteriodes abscessus* infection

AUTHORS: Emily C Maggioncalda[1], Elizabeth Story-Roller[1], Julian Mylius[2], Peter Illei[3,4], Randall J. Basaraba[2], Gyanu Lamichhane*[1]

AFFILIATIONS: 1) Division of Infectious Diseases, School of Medicine, Johns Hopkins University, Baltimore, MD, United States 2) Department of Microbiology, Immunology, and Pathology, College of Veterinary Medicine and Biomedical Sciences, Colorado State University, Fort Collins, Colorado, United States 3) The Sidney Kimmel Comprehensive Cancer Center, Johns Hopkins University School of Medicine, Baltimore, Maryland, United States 4) Department of Pathology, Johns Hopkins University School of Medicine, Baltimore, Maryland, United States

SUPPLEMENTAL METHODS

Liver and Spleen CFU Enumeration

Whole spleen from sacrificed mice were homogenized in 1x PBS and cultured on Middlebrook 7H11 agar to determine *M. abscessus* CFU. For liver CFU enumeration approximately one-fifth of the liver was sampled and homogenized in 1xPBS and cultured on Middlebrook 7H11 agar. The resulting CFU value was then multiplied by five to approximate the whole liver burden before calculating the $\log_{10}$ CFU.

Histology Preparation and Analysis

For the day 0, week 1, and week 3 time-points, two mice selected at random were evaluated for pathology and CFU. For the week 5 and week 7 time-points, all mice were sampled for both CFU and pathology. In mice allocated for both CFU and pathology, after sacrifice, lungs were aseptically collected and gross images taken.

Then, one-half of the lungs were homogenized in sterile 1xPBS and cultured on selective Middlebrook 7H11 agar to determine *Mab* CFU and the other half was processed for histopathological examinations. To approximate total CFU burden in a mouse, the resultant CFU from the homogenate from each half-lung was doubled.

The other half-lungs were fixed in 10% neutral buffered formalin solution (Sigma HT501128) for a minimum of 48 hours before embedding in paraffin blocks. For unbiased histopathological assessments, a standardized section of lung was prepared and examined for presence of any lesions associated with infection, as well as presence and number of *Mab* bacilli and any associated fibrosis by staining the sections with Hematoxylin & eosin or Ziehl Nielson or and Masson Trichrome according to manufacturer's instructions.

<u>Pathology Analysis and Lesion Quantification</u>

Histology slides were by examined independently by an American College of Veterinary board-certified pathologist and by an American Board of Pathology board-certified pathologist and cytopathologist who were blinded to treatment conditions.

Lesion quantification was performed using Stereo Investigator, version 2019.1.2 (MBF Bioscience, Williston, VT USA) and a Nikon Eclipse 80i microscope. Unbiased stereology was conducted with the area fraction fractionator probe to estimate the area of lesion on a systematically determined fraction of lung tissue. Lung sections were traced at 4x objective. A systematic random sampling grid was generated and counting boxes (500 µm x 500 µm) were placed in the trace to 50 µm apart. Analysis was achieved at 20x/40x at each counting site by designating markers as either lesion or tissue.

Lung lobes with representative gross lesions were collected at necropsy and fixed in 4% paraformaldehyde in phosphate-buffered saline (PBS). Randomly selected tissue sections were embedded in paraffin and cut to 5m on a microtome. The tissue sections were mounted on glass slides, deparaffinized and stained with hematoxylin-eosin and carbolfuchsin by the Ziehl-Neelsen method. The lung lesion areas relative to the normal tissue area were quantified by a stereology-based method, referred to as the area fraction fractionator. The stereology work station consisted of a Nikon 80i research microscope equipped for bright-field and fluorescence microscopy with chromatic aberration-free infinity (CFI) objectives(2/0.01 Plan Apo,4/0.2 Plan Apo,10/0.30 Plan Fluor,20/0.75 Plan Apo,40/0.75 Plan Fluor,100/1.40 Plan Apo), a three-axis computer-controlled stepping stage with linear grid encoders, az-axis motorized specimen stage for automated sampling, a personal computer with a frame grabber board, a color digital camera, a 24-in. monitor, and stereology and virtual slice zoomify software (StereoInvestigator, version 2019.1.2; MBF Bioscience, Williston, VT). The lung and lesion areas on representative hematoxylin-eosin-stained sections were determined. The area of inflammation relative to the area of normal tissue parenchyma was estimated from representative lung sections evaluated at 20X magnification. A total of 15 to 20 fields were randomly selected by the computer, and a counting frame (500 μm x 500 μm) containing probe points with a grid spacing of 50 μm was used to define the areas of interest (lesions and normal lung parenchyma). The data are expressed as the mean ratio of the lesion area to the normal lung area for all the animals within a treatment group.

Statistical Analysis

The CFU data obtained from the biapenem intranasal treatment experiment were evaluated using one-way ANOVA with a post-hoc Tukey's Multiple Comparison test.
